# Clinicopathological Spectrum, Surgical Management, and Outcomes of Uncommon Surgically Managed Small Intestinal Disorders: A Six-Year Retrospective Observational Study From a Tertiary Care Center

**DOI:** 10.7759/cureus.113385

**Published:** 2026-07-26

**Authors:** Rajesh K Nanjundaiah, Vinod B N Kumar, Naveen Narayan, Nisha Manjunatha, Chaitanya N S Prabhu

**Affiliations:** 1 General Surgery, Adichunchanagiri Institute of Medical Sciences, B G Nagara, IND; 2 General Surgery, Dr. B.R. Ambedkar Medical College, Bengaluru, IND; 3 Plastic Reconstructive and Aesthetic Surgery, Adichunchanagiri Institute of Medical Sciences, B G Nagara, IND

**Keywords:** arteriovenous malformation, crohn’s disease, gastrointestinal stromal tumor, meckel’s diverticulum, midgut volvulus, neuroendocrine tumor, small bowel pathology, surgical outcomes

## Abstract

Background

The present study focuses on uncommon surgically managed disorders of the small intestine. Although several of these entities, such as gastrointestinal stromal tumors, Crohn’s disease requiring surgery, and symptomatic Meckel’s diverticulum, are not classified as rare diseases by epidemiological definitions, they represent infrequently encountered indications for operative management at individual centers. Evaluating these surgically managed conditions together provides insight into diagnostic challenges, operative decision-making, and postoperative outcomes encountered in routine surgical practice.

Methodology

A retrospective observational study was conducted including all patients with uncommon surgically managed small intestinal disorders between January 2020 and December 2025. Clinical, operative, histopathological, and postoperative outcome data were extracted from institutional medical records. Patients were followed through outpatient records and hospital databases until the last available follow-up (median = 24 months; range = 3-36 months). Continuous variables were analyzed using Student’s t-test or Mann-Whitney U test, and categorical variables using the chi-square test or Fisher’s exact test, as appropriate. Descriptive statistics with 95% confidence intervals (CIs) were used to summarize clinical outcomes.

Results

In total, 34 patients were included. The mean age was 46.7 ± 16.4 years, and 70.6% were male (24/34; 95% CI: 52.5%-84.9%). Gastrointestinal stromal tumors were the most common pathology (26.5%; 95% CI: 12.9%-44.4%). Contrast-enhanced computed tomography was performed in all patients (34/34, 100%) and demonstrated the highest radiological-pathological concordance, with the preoperative radiological impression corresponding to the final operative and histopathological diagnosis in 31 of 34 patients (91.2%). Emergency surgery was required in 41.2% of patients (14/34; 95% CI: 24.6%-59.3%). Postoperative morbidity occurred in 11.8% (4/34; 95% CI: 3.3%-27.5%), with no postoperative mortality. During a mean follow-up of 21.4 ± 8.6 months, symptom resolution occurred in 94.1% (32/34; 95% CI: 80.3%-99.3%), disease recurrence in 5.9% (2/34; 95% CI: 0.7%-19.7%), diagnosis-specific follow-up outcomes in 94.1% (95% CI: 80.3%-99.3%), and overall survival in 100% (95% CI: 89.7%-100%).

Conclusions

Uncommon surgically managed small intestinal disorders encompass a heterogeneous group of congenital, inflammatory, vascular, and neoplastic conditions that present substantial diagnostic and therapeutic challenges. Timely diagnosis and definitive surgical management are associated with favorable postoperative outcomes; however, disease-specific interpretation remains important because of the heterogeneity of the cohort.

## Introduction

Diseases of the small intestine account for only 1-4% of gastrointestinal disorders despite comprising nearly 75% of the length and over 90% of the mucosal surface area of the gastrointestinal tract. Owing to anatomical inaccessibility, nonspecific symptomatology, and low prevalence, diagnosis often remains delayed despite significant advances in imaging and endoscopic technologies [1-4].

Uncommon surgically managed disorders of the small intestine encompass a heterogeneous group of congenital, inflammatory, vascular, and neoplastic conditions, including gastrointestinal stromal tumors (GISTs), neuroendocrine tumours (NETs), Crohn’s disease-related strictures, Meckel’s diverticulum, midgut volvulus, and arteriovenous malformations (AVMs). Although these entities differ in their underlying pathophysiology and epidemiology and are not uniformly classified as rare diseases, they share several clinically important features. They often present with nonspecific symptoms such as abdominal pain, intestinal obstruction, gastrointestinal bleeding, anemia, or acute abdomen, making diagnosis challenging. Furthermore, many ultimately require definitive surgical intervention involving bowel resection, restoration of intestinal continuity, and histopathological confirmation. Evaluating these surgically managed disorders together provides a practical overview of the diagnostic pathways, operative strategies, perioperative outcomes, and postoperative results encountered in tertiary referral surgical practice while acknowledging the heterogeneity of the included conditions [5-8].

GISTs represent the most common mesenchymal neoplasms of the gastrointestinal tract and arise from interstitial cells of Cajal. Small bowel NETs are uncommon but possess significant metastatic potential. Adult midgut volvulus remains a rare surgical emergency with substantial mortality when bowel ischemia develops. Similarly, complicated Crohn’s disease, symptomatic Meckel’s diverticulum, and vascular malformations often necessitate surgical management despite advances in medical therapy and endoscopic intervention [9-14].

Most published literature addresses these disorders individually, whereas studies evaluating the collective experience of surgically managed uncommon small intestinal conditions are limited. A broader surgical perspective may help identify common diagnostic challenges, operative principles, and postoperative outcome patterns across these conditions while recognizing their disease-specific differences.

Most available literature focuses on individual disease entities. Comprehensive studies evaluating the entire spectrum of uncommon surgically managed small intestinal disorders remain scarce, particularly from developing countries. The present study aims to evaluate the clinicopathological characteristics, operative management, and outcomes of uncommon surgically managed small intestinal disorders at a high-volume tertiary referral center.

## Materials and methods

Study design and setting

This single-center, retrospective observational cohort study was conducted in the Department of General Surgery, Adichunchanagiri Institute of Medical Sciences and Hospital, B.G. Nagara, Karnataka, India. Consecutive patients who underwent surgical intervention for uncommon small intestinal disorders between January 2020 and December 2025 were included. Institutional Ethics Committee approval was obtained before commencement of the study, and the requirement for informed consent was waived because of the retrospective design and use of anonymized patient records (approval number: AIMS/IEC/266/2026). The study was designed, conducted, and reported in accordance with the Strengthening the Reporting of Observational Studies in Epidemiology (STROBE) Statement, and the completed STROBE checklist has been submitted as supplementary material.

Study population

All consecutive patients diagnosed with uncommon small intestinal pathologies requiring operative management during the study period were screened for eligibility. A universal sampling strategy was adopted, whereby all eligible patients treated during the study period were included. The study focused on uncommon surgically managed disorders of the small intestine, including GISTs, recurrent GISTs, NETs, Crohn’s disease requiring surgical intervention, midgut volvulus, Meckel’s diverticulum, AVMs, and other uncommon small bowel lesions encountered in routine surgical practice.

Inclusion and exclusion criteria

Patients of any age or sex who underwent definitive surgical treatment for an uncommon small intestinal pathology during the study period were eligible for inclusion. Only patients with complete clinical documentation, operative records, imaging findings, histopathological confirmation wherever applicable, and available postoperative follow-up data were included in the final analysis. Histopathological diagnosis was considered mandatory for all resected neoplastic, inflammatory, and vascular lesions to ensure diagnostic accuracy.

Patients managed conservatively without surgical intervention were excluded from the study. Cases with incomplete medical records, missing operative notes, unavailable histopathological reports, or inadequate follow-up data preventing assessment of postoperative outcomes were also excluded. Patients whose hospital records were lost or contained insufficient information for meaningful analysis were not considered for inclusion.

Data collection

Patient data were retrieved from multiple institutional sources, including operation theater registers, inpatient surgical records, hospital information systems, histopathology databases, and medical records department archives. A standardized data collection proforma was used to ensure uniform extraction of variables.

Demographic data included age, sex, body mass index, comorbid illnesses, and previous abdominal surgeries. Clinical variables recorded included presenting symptoms, duration of symptoms, history of bowel obstruction, gastrointestinal bleeding, and previous interventions. Diagnostic parameters comprised laboratory investigations, tumor markers when applicable, ultrasonography, computed tomography (CT), magnetic resonance imaging (MRI), endoscopic findings, and colonoscopic findings.

Operative variables included urgency of surgery (emergency versus elective), type of surgical procedure performed, length of bowel resected, stoma creation, operative duration, estimated blood loss, and requirement for intensive care unit admission. Histopathological variables included final diagnosis, lesion size, tumor grade, lymph node status, margin status, mesenteric involvement, lymphovascular invasion, and TNM staging where applicable.

Because of the retrospective nature of the study, standardized perioperative risk stratification measures such as the American Society of Anesthesiologists (ASA) Physical Status Classification were not consistently documented across the entire study period and therefore could not be included in the analysis. Instead, baseline health status was characterized using demographic variables, body mass index, and documented comorbid illnesses.

Surgical management

The choice of operative procedure was individualized according to the underlying pathology, disease extent, patient condition, and intraoperative findings. Segmental bowel resection with restoration of intestinal continuity was the preferred surgical approach whenever feasible. Stoma creation was performed selectively in patients with compromised nutritional status, severe contamination, questionable bowel viability, or when primary anastomosis was considered unsafe. All surgical specimens were submitted for detailed histopathological examination.

**Figure 1:**
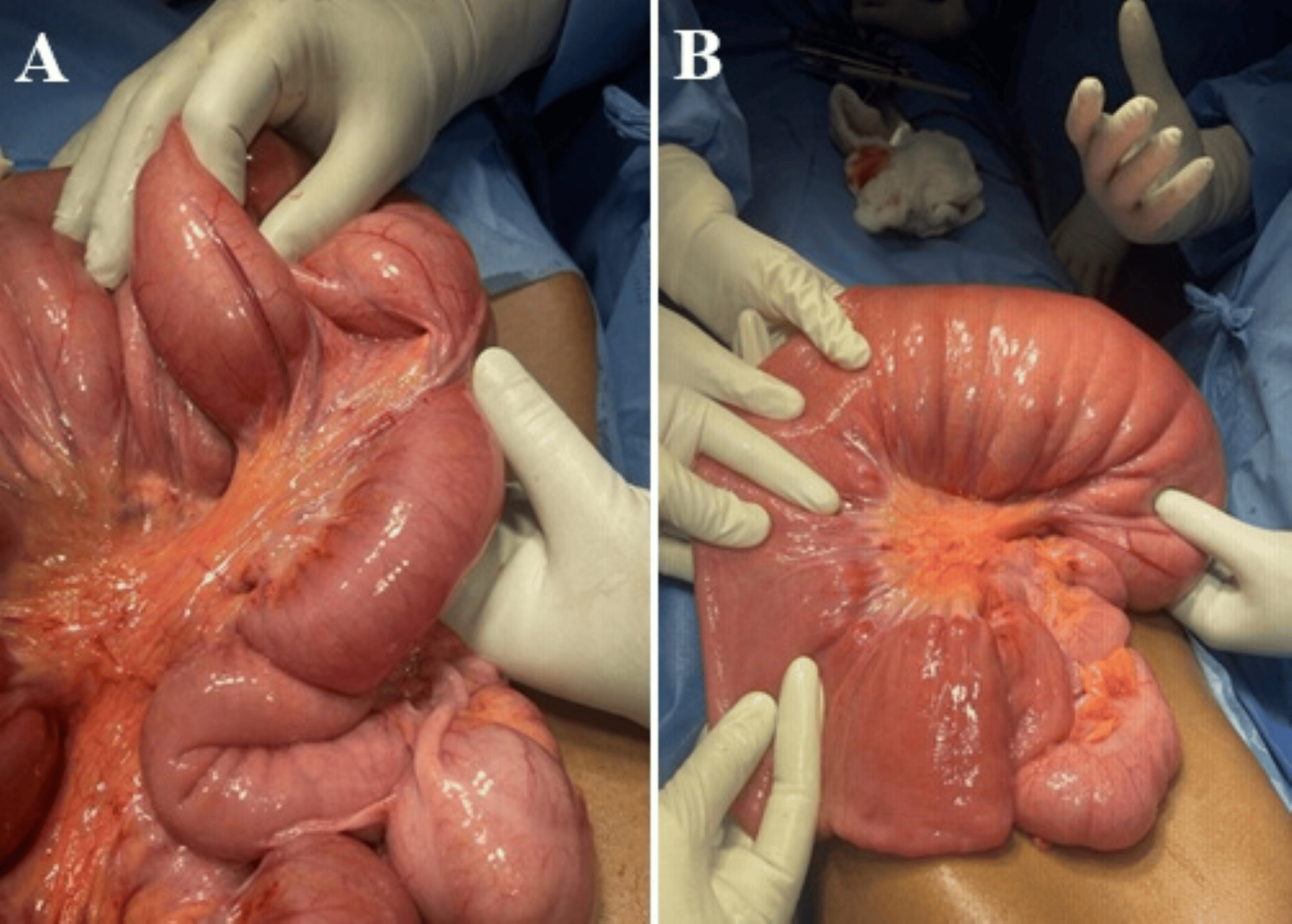
Midgut volvulus. Intraoperative photograph demonstrating midgut volvulus with dilated proximal small bowel loops and torsion around a congenital mesenteric band causing small bowel obstruction (A). Following adhesiolysis, the congenital mesenteric band is seen at the root of the mesentery with markedly dilated jejunal loops before bowel resection and jejunoileal anastomosis (B).

The intraoperative findings of adult midgut volvulus with the congenital mesenteric band and the operative appearance following adhesiolysis are illustrated in Figure 1.

**Figure 2:**
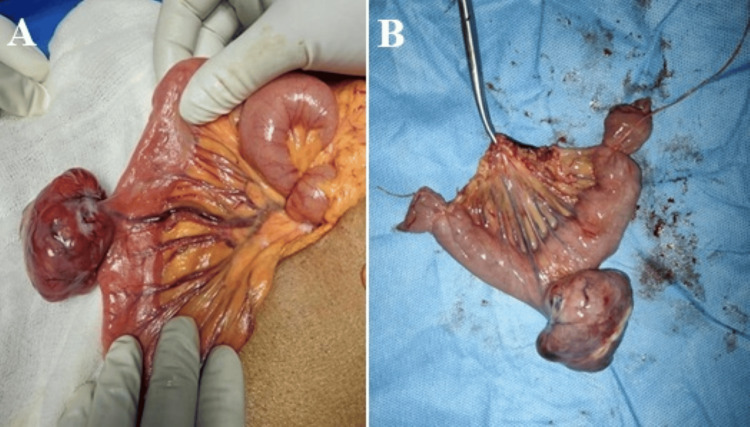
Ileal gastrointestinal stromal tumor. Intraoperative image demonstrating a large exophytic gastrointestinal stromal tumor (GIST) arising from the proximal ileum (A). Resected ileal specimen containing the tumor following en bloc segmental ileal resection with adequate surgical margins (B).

Figure 2 demonstrates the intraoperative appearance of a large ileal GIST and the resected specimen following segmental bowel resection.

**Figure 3:**
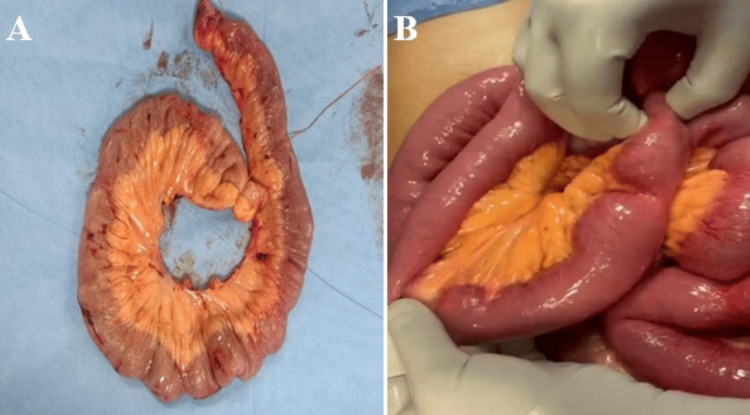
Crohn’s disease. Resected jejunal and ileal segment showing multiple strictures with transmural inflammatory changes characteristic of Crohn’s disease (A). Intraoperative photograph demonstrating multiple skip lesions and strictures involving the small intestine with mesenteric thickening (B).

Representative intraoperative and gross pathological findings of Crohn’s disease with multiple strictures are shown in Figure 3.

**Figure 4:**
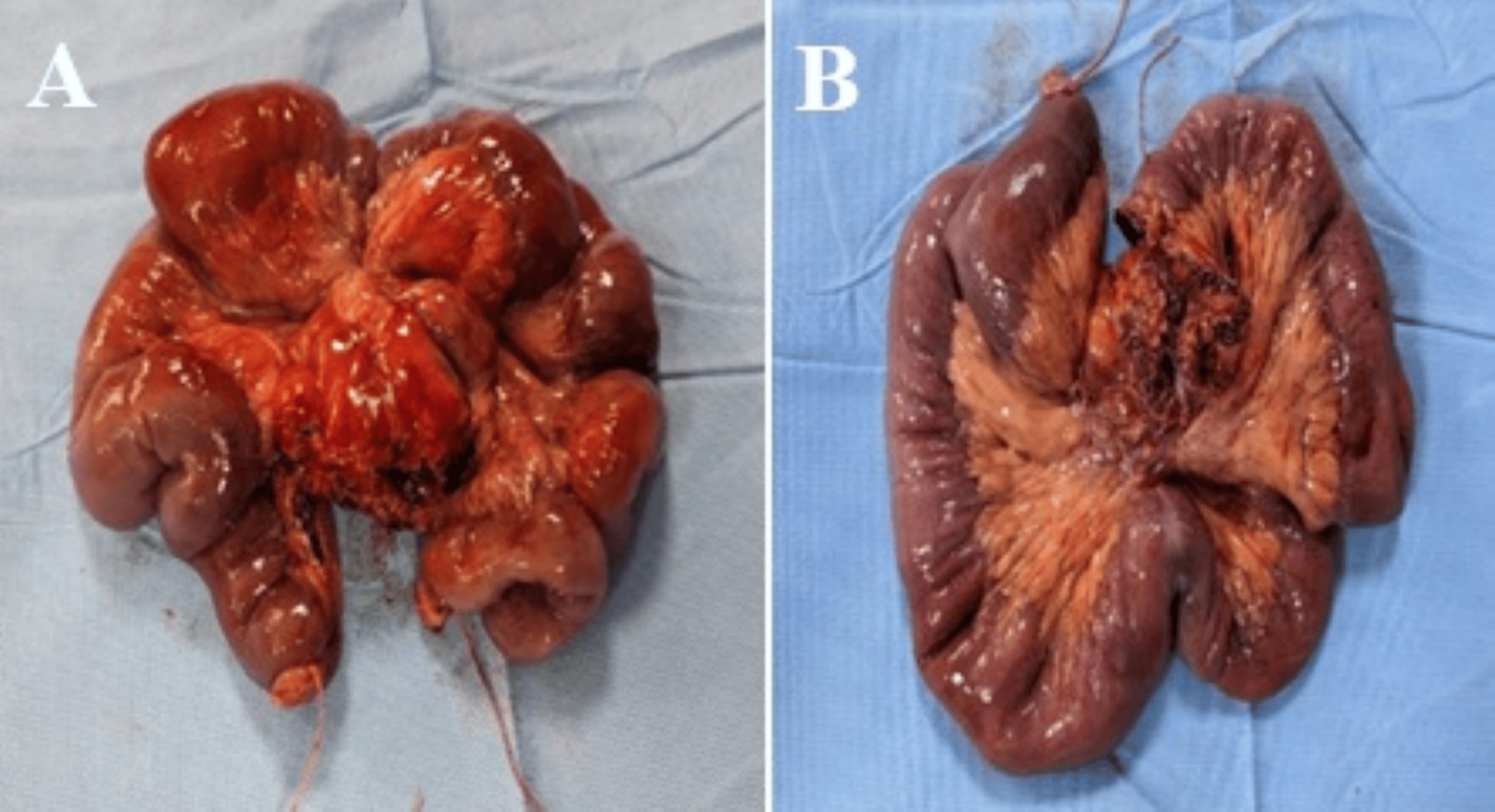
Neuroendocrine tumor. Resected specimen demonstrating an ileal neuroendocrine tumor with associated mesenteric mass following segmental bowel resection (A). Gross pathological specimen showing the mesenteric tumor with surrounding small bowel after oncological resection (B).

The operative findings and resected specimen of an ileal NET with an associated mesenteric mass are presented in Figure 4.

**Figure 5:**
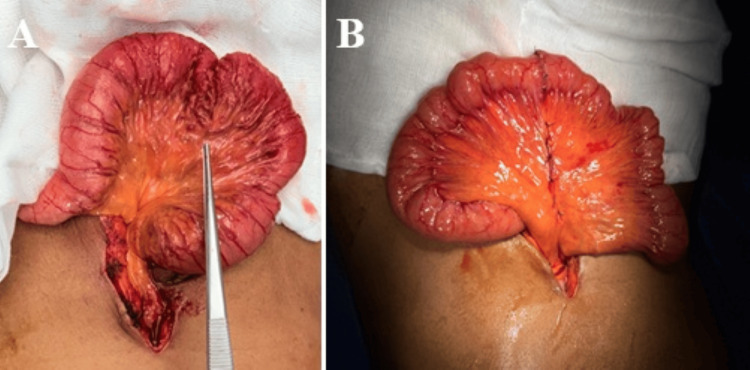
Jejunal arteriovenous malformation. Intraoperative photograph showing the vascular malformation involving the jejunal mesentery with prominent congested vessels (A). Exteriorized jejunal segment demonstrating the localized arteriovenous malformation prior to segmental resection (B).

Figure 5 illustrates the intraoperative appearance of a jejunal AVM and the affected bowel segment before resection.

**Figure 6:**
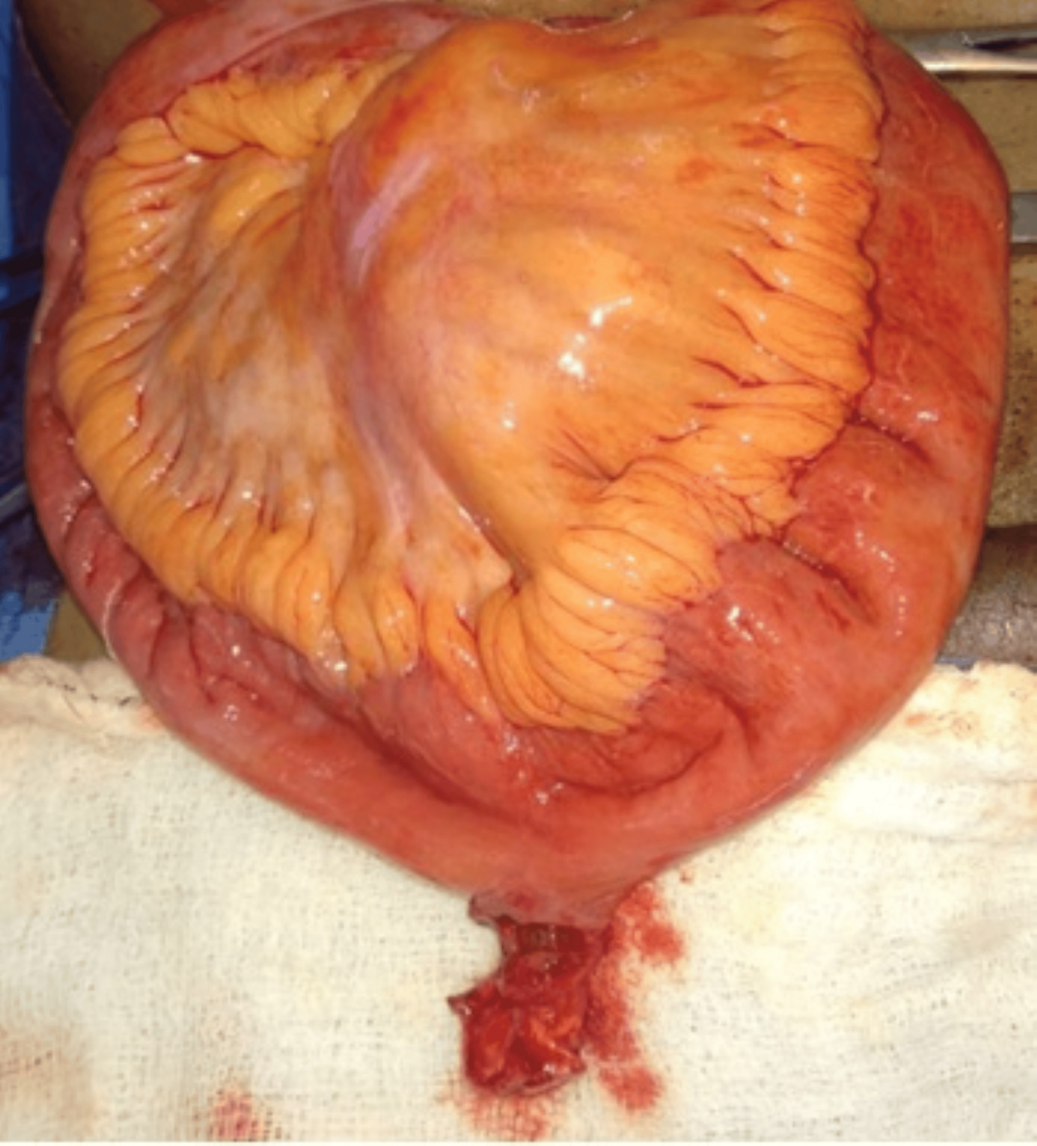
Meckel’s diverticulum. Intraoperative photograph demonstrating an inflamed Meckel’s diverticulum arising from the antimesenteric border of the distal ileum with surrounding inflammatory changes before segmental ileal resection.

The characteristic intraoperative appearance of an inflamed Meckel’s diverticulum is shown in Figure 6.

**Figure 7:**
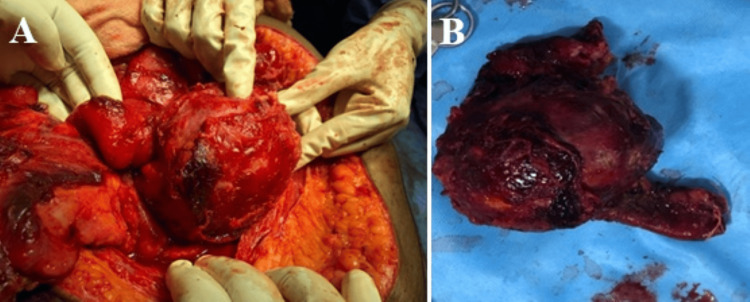
Recurrent gastrointestinal stromal tumor. Intraoperative image showing recurrent mesenteric gastrointestinal stromal tumor (GIST) involving the distal ileum with infiltration into adjacent structures (A). Gross specimen following radical en bloc resection demonstrating the recurrent high-grade GIST with attached small bowel segment (B).

Figure 7 demonstrates the operative findings of recurrent GIST and the corresponding resected specimen following radical en bloc resection.

Outcome measures

The primary outcome measures included postoperative morbidity, mortality, length of hospital stay, symptom resolution, disease recurrence, diagnosis-specific follow-up outcomes, and overall survival. Follow-up information was obtained from outpatient clinic records, hospital electronic medical records, imaging reports, histopathology records, and subsequent hospital admissions.

Postoperative surveillance was performed according to the underlying pathology and prevailing institutional clinical practice rather than a uniform study-specific protocol because of the retrospective design. Patients with GISTs underwent routine clinical review with contrast-enhanced CT of the abdomen and pelvis at intervals determined by tumor risk category and treating surgeon preference, generally every 6-12 months. Additional imaging was performed whenever recurrence was clinically suspected.

Patients with NETs underwent postoperative clinical review with cross-sectional imaging (contrast-enhanced CT or MRI) based on tumor stage and treating clinician discretion. Biochemical markers such as chromogranin A or 24-hour urinary 5-hydroxyindoleacetic acid were reviewed when available but were not consistently obtained in all patients and therefore were not included in the study analysis.

In patients with Crohn’s disease, recurrence was defined as recurrent clinical symptoms requiring further medical escalation or repeat surgical intervention, supported by radiological, endoscopic, or operative findings where available.

Disease recurrence was defined as radiological, histopathological, or clinically confirmed reappearance of the index disease following definitive surgical treatment. Diagnosis-specific follow-up outcomes were assessed from the date of surgery until documented recurrence or the last available follow-up.

Clinical outcomes during follow-up were assessed using outpatient follow-up records. Clinical improvement was defined as documented resolution or substantial improvement of the patient’s principal presenting symptom(s) that prompted surgical intervention, without the need for reoperation or disease-specific readmission during follow-up. Because this retrospective cohort included heterogeneous pathological entities, no single validated disease-specific patient-reported outcome measure was applicable across all diagnoses.

Statistical analysis

Statistical analyses were performed using SPSS Statistics version 29.0 (IBM Corp., Armonk, NY, USA). Continuous variables are presented as mean ± standard deviation or median (interquartile range) according to data distribution, whereas categorical variables are expressed as frequencies and percentages. Comparisons between two groups were performed using the independent-samples Student’s t-test for continuous variables and Pearson’s chi-square test or Fisher’s exact test for categorical variables, as appropriate. For key proportions, 95% confidence intervals were calculated using the exact binomial (Clopper-Pearson) method. A two-sided p-value <0.05 was considered statistically significant.

## Results

Patient characteristics and disease spectrum

**Figure 8:**
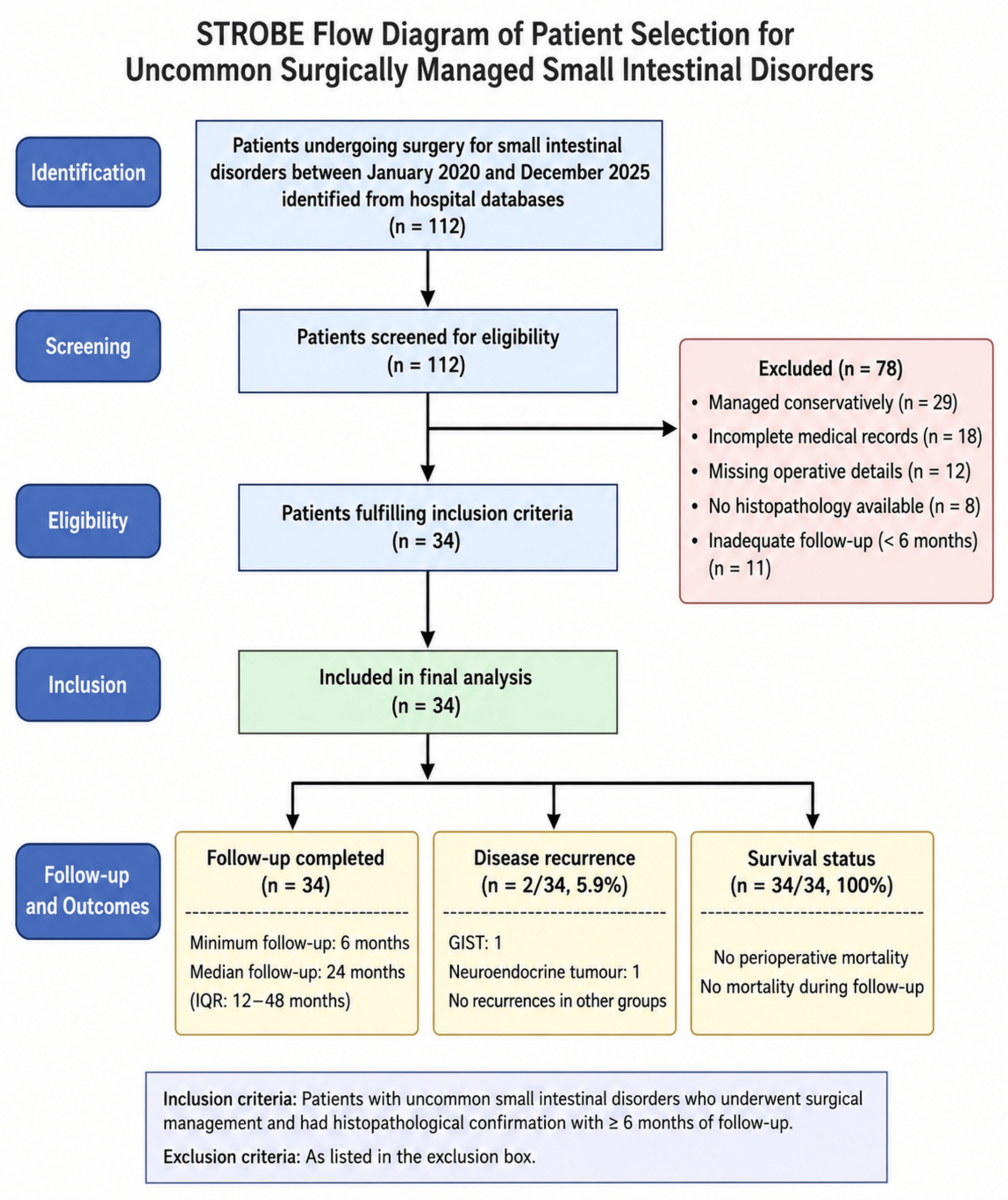
STROBE flow diagram of patient selection for uncommon surgically managed small intestinal disorders.

A total of 34 patients with uncommon surgically managed small intestinal disorders during the six-year study period fulfilled the eligibility criteria for inclusion. The participant selection process is illustrated in Figure 8.

The annual case distribution is summarized in Table 1 and demonstrated a relatively consistent referral pattern throughout the study period. The cohort comprised congenital (9/34, 26.5%), inflammatory (5/34, 14.7%), vascular (4/34, 11.8%), and neoplastic (16/34, 47.1%) disorders. Neoplastic lesions represented the largest disease category, with GISTs (9/34, 26.5%) being the most frequent individual pathology, followed by Crohn’s disease (5/34, 14.7%) and Meckel’s diverticulum (5/34, 14.7%) (Table 1).

**Table 1:** Distribution of surgically managed uncommon small intestinal disorders according to disease category and study year (2020–2025). Percentages are calculated using the total study population (n = 34). Recurrent gastrointestinal stromal tumor was analyzed separately because of its distinct biological behavior and recurrence profile.

Disease category	Pathology	2020	2021	2022	2023	2024	2025	Total, n (%)
Congenital	Meckel’s diverticulum	1	-	1	1	-	2	5 (14.7)
	Midgut volvulus	-	1	-	1	1	1	4 (11.8)
	Subtotal	1	2	-	2	1	3	9 (26.5)
Inflammatory	Crohn’s disease	-	1	1	-	2	1	5 (14.7)
	Subtotal	-	1	1	-	2	1	5 (14.7)
Vascular	Arteriovenous malformation	1	1	1	-	1	-	4 (11.8)
	Subtotal	1	1	1	-	1	-	4 (11.8)
Neoplastic	Gastrointestinal stromal tumor (GIST)	2	-	1	3	2	1	9 (26.5)
	Recurrent GIST	-	-	-	2	1	-	3 (8.8)
	Neuroendocrine tumor	-	1	1	1	-	1	4 (11.8)
	Subtotal	2	1	2	6	3	2	16 (47.1)
Overall total	-	4	5	4	8	7	6	34 (100)

The demographic profile of the study population is presented in Table 2. The mean age was 46.7 years, with patients ranging from adolescence to advanced age. A clear male predominance was observed, accounting for more than two-thirds of the cohort. Comorbid illnesses such as diabetes mellitus and hypertension were present in a minority of patients, reflecting the heterogeneous nature of the underlying diseases.

**Table 2:** Demographic characteristics of the study population (n = 34). Continuous variables are presented as mean ± standard deviation or median (interquartile range). Categorical variables are presented as frequency and percentage.

Variable	Value
Mean age (years)	46.7 ± 16.4
Median age (IQR)	49 (31–58)
Male	24 (70.6%)
Female	10 (29.4%)
BMI (kg/m²)	23.8 ± 3.2
Diabetes mellitus	6 (17.6%)
Hypertension	8 (23.5%)
Previous abdominal surgery	5 (14.7%)

Clinical presentation

Clinical presentation was highly variable and largely depended on the underlying pathology. Abdominal pain was the universal presenting symptom and was reported by all patients. Features of intestinal obstruction, including vomiting and abdominal distension, were common, whereas gastrointestinal bleeding and anemia were predominantly observed in patients with vascular malformations and Meckel’s diverticulum (Table 3).

**Table 3:** Clinical features of the study population (n = 34). Individual patients may have presented with more than one symptom; therefore, percentages may exceed 100% when combined.

Symptom	n (%)
Abdominal pain	34 (100)
Vomiting	19 (55.9)
Intestinal obstruction	15 (44.1)
Abdominal distension	12 (35.3)
Gastrointestinal bleeding/Melena	7 (20.6)
Weight loss	8 (23.5)
Fever	6 (17.6)
Anemia	8 (23.5)

Diagnostic evaluation

A variety of diagnostic modalities were utilized during patient evaluation. Contrast-enhanced CT was performed in all patients and demonstrated the highest preoperative diagnostic concordance, confirming its central role in the investigation of uncommon surgically managed small intestinal disorders. Endoscopic modalities were useful in selected cases, particularly inflammatory and bleeding disorders, while tumor markers aided diagnosis in patients with suspected NETs and GISTs (Table 4).

**Table 4:** Preoperative imaging modalities and radiological–pathological concordance. Radiological–pathological concordance was defined as agreement between the principal preoperative radiological diagnosis documented in the clinical record and the final operative and histopathological diagnosis. Because of the retrospective observational design, these values represent diagnostic concordance rather than formal measures of diagnostic accuracy, sensitivity, or specificity.

Investigation	Performed, n (%)	Concordant with final diagnosis, n (%)
Ultrasound	29 (85.3)	44.8
Contrast CT	34 (100)	91.2
MRI enterography	5 (14.7)	80.0
Upper gastrointestinal endoscopy	12 (35.3)	25.0
Colonoscopy	7 (20.6)	57.1
Tumor markers	8 (23.5)	75.0

Operative characteristics

Surgical management was individualized according to the underlying pathology and clinical presentation. Approximately two-fifths of patients required emergency surgery because of bowel obstruction, volvulus, complicated Crohn’s disease, or acute inflammatory presentations. Segmental bowel resection was performed in all patients, highlighting the definitive role of surgery in the management of these conditions. Primary intestinal continuity was restored in the majority of cases, while temporary stomas were reserved for selected high-risk patients (Table 5).

**Table 5:** Operative characteristics of the study population (n = 34). Length of hospital stay was calculated from the date of surgery until discharge. Temporary stomas included loop ileostomies, end ileostomies, and mucous fistulas.

Variable	n (%)
Emergency surgery	14 (41.2)
Elective surgery	20 (58.8)
Open surgery	34 (100)
Segmental bowel resection	34 (100)
Primary anastomosis	29 (85.3)
Temporary stoma creation	5 (14.7)
Intensive care unit admission	4 (11.8)
Mean operative time (minute)	145 ± 48
Mean blood loss (mL)	240 ± 130

Histopathological findings

Histopathological examination confirmed the diagnosis in all resected specimens. Clinical characteristics and postoperative outcomes are presented descriptively according to individual pathological diagnoses because the heterogeneous nature of the included disorders precluded statistically valid comparisons between pooled benign and neoplastic groups (Table 6). GISTs represented the largest neoplastic subgroup. Because mitotic index was not consistently available in this retrospective cohort, formal National Institutes of Health (NIH) risk stratification was not performed.

**Table 6:** Clinical outcomes according to histopathological diagnosis (n = 34). Values are expressed as mean ± standard deviation or frequency (%). Outcomes are presented descriptively according to the final histopathological diagnosis. Because the study cohort comprised biologically distinct congenital, inflammatory, vascular, and neoplastic disorders, no inferential statistical comparisons were performed between pooled disease categories. Disease recurrence was observed only in patients with recurrent gastrointestinal stromal tumor (GIST). Intensive care unit (ICU) admission occurred predominantly among patients with complex neoplastic lesions and Crohn’s disease.

Histopathological diagnosis	Patients, n	Mean length of hospital stay (days)	ICU admission, n (%)	Postoperative complication, n (%)	Disease recurrence, n (%)
Gastrointestinal stromal tumor (GIST)	9	8.1 ± 2.4	1 (11.1)	1 (11.1)	0
Recurrent GIST	3	13.0 ± 2.6	1 (33.3)	0	2 (66.7)
Crohn’s disease	5	11.8 ± 3.2	1 (20.0)	1 (20.0)	0
Neuroendocrine tumor	4	10.5 ± 2.1	1 (25.0)	0	0
Midgut volvulus	4	8.3 ± 2.0	0	0	0
Arteriovenous malformation	4	5.5 ± 1.4	0	0	0
Meckel’s diverticulum	5	6.0 ± 1.6	0	0	0

Descriptive comparison of emergency and elective surgical presentations

Patients undergoing emergency surgery demonstrated a longer median hospital stay than those managed electively. However, because emergency procedures were performed for acute presentations such as volvulus, bowel obstruction, perforation, or severe inflammatory disease, these findings are descriptive and should not be interpreted as indicating that surgical urgency independently prolonged hospitalization. (Table 7).

**Table 7:** Comparison of emergency and elective surgery. Emergency surgery included procedures performed for acute obstruction, volvulus, perforation, severe inflammation, or impending bowel ischemia.

Variable	Emergency (n = 14)	Elective (n = 20)	P-value
Mean length of stay	10.8 ± 3.4	7.3 ± 2.1	0.004
Intensive care unit admission	3 (21.4%)	1 (5.0%)	0.14
Complications	3 (21.4%)	1 (5.0%)	0.14
Mortality	0	0	—

Postoperative morbidity

Overall postoperative morbidity occurred in 4 of 34 patients (11.8%). Two patients developed superficial surgical site infections, both of which resolved with antibiotic therapy and local wound care (Clavien-Dindo Grade II). One patient developed postoperative prolonged ileus that responded to conservative management (Grade II), while another experienced postoperative pneumonia requiring intravenous antibiotics and respiratory physiotherapy (Grade II). No patient required reoperation (Grade III or higher), intensive organ support, or experienced postoperative mortality. Most postoperative complications were minor (Clavien-Dindo Grade II) and were successfully managed conservatively without reoperation or mortality (Table 8).

**Table 8:** Postoperative complications according to Clavien-Dindo classification. Clavien–Dindo Grade I complications required minimal intervention, whereas Grade II complications required pharmacological treatment. No Grade III–V complications were observed.

Complication	Number (%)	Clavien-Dindo grade	Management
Surgical site infection	2 (5.9)	II	Antibiotics and dressing changes
Postoperative ileus	1 (2.9)	II	Conservative treatment
Pneumonia	1 (2.9)	II	Antibiotics and chest physiotherapy
Reoperation	0	—	—
Mortality	0	—	—
Total	4 (11.8)	—	—

Follow-up outcomes during the study period

At the final clinical follow-up, 32 of 34 patients (94.1%) had documented resolution or substantial improvement of their principal presenting symptom(s), based on treating surgeon assessment recorded during routine outpatient follow-up. Two patients experienced persistent or recurrent disease-related symptoms, corresponding to recurrent GIST requiring further oncological management (Table 9).

**Table 9:** Follow-up outcomes. Clinical improvement was defined as the absence of radiological, clinical, or histopathological evidence of recurrent disease during follow-up.

Variable	Value
Mean follow-up (months)	21.4 ± 8.6
Symptom resolution	32 (94.1%)
Disease recurrence	2 (5.9%)
Additional surgery	5 (14.7%)
Clinical improvement	94.1%
Overall survival	100%

At a mean follow-up of 21.4 ± 8.6 months, disease recurrence occurred in two (5.9%) patients, no patient died, and no patient required reoperation. Documented clinical improvement of the presenting symptoms was observed in 32 (94.1%) patients.

Overall postoperative outcomes

The overall postoperative outcome profile demonstrated favorable surgical results. Surgical site infection occurred in only two patients, while no cases of anastomotic leak, reoperation, or mortality were recorded. These findings support the safety and effectiveness of surgical intervention for uncommon small intestinal disorders when performed in specialized centers (Table 10).

**Table 10:** Overall postoperative outcomes of the study population (n = 34). Surgical site infection was diagnosed according to standard Centers for Disease Control and Prevention (CDC) criteria.

Outcome	n (%)
Surgical site infection	2 (5.9)
Anastomotic leak	0
Reoperation	0
Mortality	0
Recurrence	2 (5.9)
Symptom resolution	32 (94.1)

Survival analysis

Because of the low number of recurrence events (n = 2) and the absence of mortality events during the available follow-up period, formal time-to-event survival modelling was not performed. Clinical outcomes are therefore reported descriptively as recurrence rates and observed survival at the last available follow-up.

At a mean follow-up of 21.4 ± 8.6 months, two (5.9%) patients experienced disease recurrence, while no deaths occurred. Owing to the limited number of recurrence events and the absence of mortality events, formal survival modelling was not undertaken.

Predictors of postoperative complications

Emergency surgery and temporary stoma creation were associated descriptively with postoperative complications in the multivariable model. However, these findings should be interpreted cautiously because standardized perioperative risk measures such as ASA Physical Status Classification were unavailable and residual confounding by baseline patient health status cannot be excluded. Although malignant pathology and advanced age demonstrated increased odds ratios, these variables did not reach statistical significance after adjustment.

Pathology-specific surgical outcomes

Outcome analysis according to individual pathology demonstrated favorable results across all disease categories. Patients with AVMs and Meckel’s diverticulum experienced the shortest hospital stays, whereas recurrent GIST and Crohn’s disease were associated with prolonged hospitalization because of disease complexity and the need for staged surgical management (Table 11).

**Table 11:** Pathology-specific surgical outcomes. Recurrence was observed exclusively in the recurrent gastrointestinal stromal tumor (GIST) subgroup. Intensive care unit (ICU) admissions occurred primarily among patients with malignant disease and complex Crohn’s disease.

Pathology	Length of stay	Intensive care unit	Complication	Recurrence
Gastrointestinal stromal tumor (GIST)	8.1	1	1	0
Recurrent GIST	13.0	1	0	2
Crohn’s disease	11.8	1	1	0
Neuroendocrine tumor	10.5	1	0	0
Volvulus	8.3	0	0	0
Arteriovenous malformation	5.5	0	0	0
Meckel’s diverticulum	6.0	0	0	0

Diagnosis-specific follow-up outcomes

Follow-up outcomes were reported according to the underlying pathology rather than a uniform disease-free survival endpoint. During the available follow-up period, two patients with recurrent GIST experienced further disease recurrence. No recurrence was documented among patients with primary GIST or NET. Patients undergoing surgery for Meckel’s diverticulum, midgut volvulus, and AVMs remained free of disease-related readmission or reoperation during follow-up. Among patients with Crohn’s disease, no clinically documented postoperative relapse requiring reoperation occurred during the follow-up period. (Table 12).

**Table 12:** Diagnosis-specific follow-up outcomes.

Diagnosis	Patients (n)	Disease-specific follow-up outcome
Primary gastrointestinal stromal tumor (GIST)	9	No documented recurrence during follow-up
Recurrent GIST	3	Two patients experienced further recurrence
Neuroendocrine tumor	4	No documented recurrence
Crohn’s disease	5	No relapse requiring reoperation during follow-up
Midgut volvulus	4	No recurrent volvulus or disease-related readmission
Meckel’s diverticulum	5	No disease-related recurrence following resection
Arteriovenous malformation	4	No recurrent bleeding requiring intervention

## Discussion

This study represents one of the largest single-center Indian series evaluating the entire spectrum of uncommon surgically managed small intestinal disorders. Several important observations emerge.

First, neoplastic lesions constituted nearly half of all cases, with GISTs representing the predominant pathology. This finding is consistent with contemporary epidemiological studies demonstrating that GISTs are the most frequently encountered mesenchymal neoplasms of the gastrointestinal tract [15,16].

Second, abdominal pain remained the universal presenting symptom, highlighting the nonspecific nature of clinical presentation. Similar observations have been reported by Paulsen et al. [17] and Hara et al. [13], who emphasized the diagnostic delay frequently encountered in small bowel disorders.

Third, contrast-enhanced CT demonstrated high radiological-pathological concordance within this retrospective cohort. However, because imaging interpretations were assessed retrospectively and not under a predefined diagnostic accuracy protocol, these findings should not be interpreted as formal measures of diagnostic accuracy [13,17].

Emergency surgery was necessary in over 40% of patients, largely driven by volvulus, obstructive Crohn’s disease, complicated Meckel’s diverticulum, and advanced NETs. Importantly, emergency surgery was associated descriptively with increased postoperative morbidity and prolonged hospitalization.

Despite the complexity of pathology, outcomes were highly favorable. Overall morbidity remained low (11.8%), no anastomotic leaks occurred, and mortality was absent. These results compare favorably with previously reported morbidity rates of 15-30% in complex small bowel surgery [7,10].

During the available follow-up period, no recurrence was observed among patients undergoing surgery for primary GISTs, whereas two patients in the recurrent GIST subgroup experienced further disease recurrence. Given that patients in the recurrent GIST cohort had already demonstrated recurrent disease before inclusion, these findings should be interpreted descriptively rather than as evidence of increased biological aggressiveness. The absence of recurrence among primary GISTs is encouraging; however, the relatively short follow-up period and small sample size preclude conclusions regarding long-term oncological control, as late recurrence is well recognized in GIST [18,19].

The 94.1% diagnosis-specific follow-up outcomes and 100% overall survival observed during follow-up demonstrate that timely diagnosis combined with definitive surgical intervention can achieve favorable early and intermediate-term outcomes even in complex small bowel disorders.

Detailed subgroup analysis by pathology

Subgroup analysis demonstrated distinct clinicopathological and outcome characteristics across the various disease entities included in the study. GISTs, including recurrent GISTs, constituted the largest pathological subgroup and represented the majority of malignant lesions. These patients were generally older, underwent predominantly elective resections, and exhibited larger lesion sizes compared with benign pathologies. Patients with recurrent GIST experienced further recurrence during follow-up, reflecting the recurrent nature of this subgroup rather than serving as independent evidence of biological aggressiveness. Despite this, overall disease control remained favorable following complete surgical excision. Mitotic index and other variables required for formal NIH/Armed Forces Institute of Pathology risk stratification of GISTs were not consistently available because of the retrospective design. Consequently, GISTs were reported descriptively as neoplastic lesions without assignment to malignant risk categories.

Patients with Crohn’s disease represented a younger population and commonly presented with acute intestinal obstruction secondary to multiple strictures and inflammatory bowel involvement. This subgroup had one of the highest rates of emergency surgery and temporary stoma creation because of the complexity of disease presentation. Nevertheless, postoperative outcomes were favorable, with restoration of bowel continuity in selected patients and sustained symptom resolution during follow-up when combined with appropriate medical therapy.

NETs were predominantly encountered in elderly patients and frequently demonstrated mesenteric involvement and regional lymph node metastasis at presentation. Radical oncological resection with mesenteric lymphadenectomy resulted in satisfactory disease control, and no recurrence was documented during the follow-up period. Similarly, patients with midgut volvulus typically presented as surgical emergencies with features of bowel obstruction. Prompt operative intervention prevented bowel ischemia and resulted in favorable postoperative recovery without recurrence.

Patients with jejunal AVMs commonly presented with chronic abdominal pain, occult gastrointestinal bleeding, or anemia. Segmental bowel resection provided definitive treatment, resulting in complete symptom resolution and absence of recurrent bleeding during follow-up. Meckel’s diverticulum was predominantly observed in younger adults and usually presented with acute inflammatory symptoms mimicking appendicitis or small bowel obstruction. Surgical resection was curative in all cases, with short hospital stays, minimal postoperative morbidity, and no evidence of recurrence.

Overall, pathology-specific outcome analysis demonstrated acceptable surgical results across all disease categories. Patients with AVMs and Meckel’s diverticulum experienced the shortest hospital stays and most rapid recovery, whereas recurrent GIST and Crohn’s disease were associated with relatively prolonged hospitalization because of disease complexity, need for staged procedures, and requirement for long-term surveillance. Favorable diagnosis-specific follow-up outcomes were observed during the available follow-up period. However, because the cohort included biologically distinct neoplastic, inflammatory, congenital, vascular, and obstructive disorders, disease-specific outcomes were reported descriptively rather than using a common disease-free survival endpoint.

The comparison between emergency and elective surgery is subject to confounding by indication. Patients requiring emergency intervention generally presented with more advanced or complicated disease, including bowel obstruction, volvulus, ischemia, or perforation, which inherently carries a greater risk of postoperative morbidity and prolonged hospitalization. As standardized measures of disease severity were unavailable, no adjustment for baseline clinical severity could be performed. Consequently, these findings should be interpreted as descriptive associations rather than evidence of an independent effect of surgical urgency on outcomes.

Clinical improvement was determined from routine follow-up documentation rather than standardized disease-specific patient-reported outcome measures. Given the heterogeneous nature of the included disorders, validated outcome instruments applicable across all pathologies were unavailable. Consequently, clinical outcomes during follow-up should be interpreted as descriptive assessments of postoperative status rather than standardized measures of symptom burden or quality of life.

The mean follow-up duration of 21.4 months permits assessment of early postoperative and intermediate-term outcomes but is insufficient to evaluate the long-term recurrence patterns of GISTs, NETs, or Crohn’s disease, all of which may recur several years after surgery. Accordingly, the reported disease-free and overall survival rates should be interpreted as outcomes observed during the available follow-up period rather than definitive long-term oncological or disease-specific results.

The small number of postoperative complications precluded reliable multivariable regression modelling. Consequently, the study was not designed or powered to identify independent predictors of postoperative morbidity, and the findings should be interpreted descriptively. Larger multicenter cohorts will be required for robust risk factor analyses.

Benchmarking against published literature

When benchmarked against landmark international studies, outcomes in the present cohort compare favorably despite the heterogeneous pathology spectrum. The Mayo Clinic [20] experience involving 1,476 patients with Meckel’s diverticulum reported symptomatic disease in only 16% of cases, whereas all Meckel’s diverticula in our study were symptomatic and surgically managed with satisfactory outcomes. Similarly, the NET subgroup demonstrated recurrence-free survival comparable to outcomes reported in NANETS consensus datasets [21,22]. Among patients with GISTs, recurrence was limited to recurrent high-risk lesions, supporting contemporary European Society for Medical Oncology recommendations advocating complete surgical excision and adjuvant imatinib therapy [23,24]. Crohn’s disease patients underwent surgery for advanced stricture disease but achieved sustained symptom resolution without recurrence during follow-up [25-27] (Table 13).

**Table 13:** Benchmarking of current study against major published series. Literature benchmarks were derived from landmark studies including the Mayo Clinic Meckel’s diverticulum series, NANETS neuroendocrine tumor (NET) guidelines, European Society for Medical Oncology (ESMO) gastrointestinal stromal tumor (GIST) cohorts, and contemporary Crohn’s disease surgical studies.

Parameter	Current study (n = 34)	Mayo Clinic Meckel series (Park et al.) [20]	NANETS midgut NET studies [21,22]	ESMO/European GIST cohorts [23,24]	Contemporary Crohn’s surgical cohorts [25-27]
Study population	Uncommon surgically managed small intestinal disorders	Meckel’s diverticulum	Small bowel NETs	GIST	Crohn’s disease
Sample size	34	1,476	Large multicenter consensus datasets	Multicenter European cohorts	Large tertiary referral cohorts
Mean age (years)	46.7 ± 16.4	Predominantly adults	60–65	55–65	25–40
Male sex (%)	70.6%	~75% symptomatic cases	55–60%	50–60%	45–55%
Abdominal pain (%)	100%	Variable	Common	Common	Common
Obstruction (%)	44.1%	Major adult presentation	Frequent advanced presentation	Less common	Common
GI bleeding (%)	20.6%	Most common symptomatic adult presentation	May occur	Rare	Uncommon
Surgical treatment (%)	100%	100% resected cases	Surgical resection cornerstone	Complete surgical excision cornerstone	Surgery for complicated disease
Recurrence (%)	5.9%	Very low after resection	Variable by stage	10–40% depending on risk	20–40% long-term
Overall morbidity (%)	11.8%	Low	10–20%	10–25%	20–35%
Overall survival (%)	100%	Favourable	5-year survival often >80–90% for localized disease	Favorable after complete resection and adjuvant therapy	Favorable but recurrent disease common

Collectively, these findings suggest that timely diagnosis, multidisciplinary evaluation, and definitive surgical management can achieve outcomes comparable to those reported by major international centers despite the rarity and complexity of these disorders. Despite managing a heterogeneous group of uncommon surgically managed small intestinal disorders, postoperative morbidity remained low, recurrence rates were minimal, and overall survival was favorable.

Strengths and limitations

The present study possesses several notable strengths. To our knowledge, it represents one of the few contemporary Indian series evaluating the entire spectrum of uncommon surgically managed small intestinal disorders within a single cohort. Unlike previous reports that have largely focused on individual disease entities, this study provides a comprehensive overview of congenital, inflammatory, vascular, and neoplastic small bowel disorders encountered in routine surgical practice. All patients underwent definitive surgical management with histopathological confirmation of diagnosis whenever applicable, ensuring diagnostic accuracy and uniformity of outcome assessment. The study also incorporates detailed clinicopathological characterization, operative variables, assessment of emergency versus elective surgery, and clinical outcomes during follow-up. Furthermore, benchmarking against major international studies enhances the external validity of the findings and allows meaningful comparison with established global literature.

Nevertheless, several limitations should be acknowledged. An important limitation of this study is the heterogeneous nature of the included disorders. Although several conditions such as symptomatic Meckel’s diverticulum, Crohn’s disease requiring surgery, and GISTs are uncommon indications for operative management, they are not classified as rare diseases according to formal epidemiological definitions. Accordingly, the findings should be interpreted as describing the outcomes of a heterogeneous cohort of surgically managed uncommon small intestinal disorders rather than a single epidemiologically rare disease entity. The retrospective design inherently carries the risk of selection bias, information bias, and incomplete documentation. The relatively small sample size reflects the rarity of these conditions and limits the statistical power of subgroup analyses and multivariable modelling. The heterogeneous nature of the included pathologies may restrict direct comparisons between disease groups and may introduce confounding factors related to disease-specific biological behavior and treatment strategies. In addition, the study was conducted at a single tertiary referral center, which may limit the generalizability of the findings to other healthcare settings. Although follow-up was available for all patients, the duration of follow-up may not have been sufficient to fully evaluate late recurrence patterns, particularly in malignant neoplasms such as GISTs and NETs. Therefore, larger multicenter studies with longer follow-up durations are warranted to validate these findings and further define prognostic factors influencing outcomes in uncommon surgically managed small intestinal disorders. Another limitation is the absence of a standardized surveillance protocol across all disease categories. Because this was a retrospective study including heterogeneous pathologies, postoperative follow-up reflected routine clinical practice rather than a predefined study protocol. Consequently, imaging frequency, biochemical surveillance, and methods of recurrence detection varied according to disease type and clinical indication. This variability may have resulted in under-detection of asymptomatic recurrence, particularly among patients with neoplastic disorders, and the reported recurrence rate should therefore be interpreted cautiously. Its retrospective observational design precludes causal inference and is subject to the inherent limitations of medical record review. The absence of a control group limits comparative evaluation of treatment strategies. As a single-institution study conducted at a tertiary referral center, the findings may be influenced by referral and selection bias and may not be fully generalizable to other practice settings. In addition, the relatively small sample size and limited follow-up restrict the assessment of uncommon outcomes and long-term disease-specific prognosis. Larger prospective multicenter studies are warranted to validate these findings.

The findings of the present study have important implications for surgeons managing uncommon small bowel disorders. Early utilization of contrast-enhanced CT and timely referral to specialized centers may facilitate diagnosis and reduce emergency presentation. Furthermore, definitive surgical resection can achieve satisfactory disease control across a broad range of uncommon small bowel pathologies.

## Conclusions

Uncommon surgically managed small intestinal disorders continue to pose major diagnostic and therapeutic challenges. Cross-sectional imaging, particularly contrast-enhanced CT, remains the cornerstone of preoperative evaluation. Surgical resection provides definitive treatment for most lesions and results in good symptom control, low morbidity, and favorable survival during the available follow-up period. Early recognition and timely referral to specialized surgical centers are critical for optimizing outcomes in these uncommon but clinically significant diseases.
